# Evaluation of dietary food intakes and anthropometric measures in middle-aged men with aggressive symptoms

**DOI:** 10.1186/s40795-023-00730-z

**Published:** 2023-06-26

**Authors:** Behnaz Abiri, Shirin Amini, Hajar Ehsani, MohammadAli Ehsani, Parisa Adineh, Hakimeh Mohammadzadeh, Sima Hashemi

**Affiliations:** 1 Obesity Research Center, Research Institute for Endocrine Sciences, Shahid Beheshti University of Medical Sciences, Tehran, Iran; 2 Department of Nutrition, Shoushtar Faculty of Medical Sciences, Shoushtar, Iran; 3 Sidon Health Center, Sidon, Khuzestan Province Iran; 4 Sidon Education Department, Sidon, Khuzestan Province Iran; 5 Student Research Committee, Shoushtar Faculty of Medical Sciences, Shoushtar, Iran; 6 Department of Nursing, Shoushtar Faculty of Medical Sciences, Shoushtar, Iran

**Keywords:** Aggressive behavior, Diet, Fruits, High-quality protein, Middle-aged men, Vegetables

## Abstract

**Background:**

Aggression is one of the most prevalent behavioral disorders in men.

**Objective:**

This study aimed to assess the possible association between dietary intake of food groups and aggression in middle-aged married men.

**Methods:**

This case-control study included 336 participants (168 men with aggressive behaviors and 168 healthy controls) aged 35–55 years. Demographic information was collected using a socio-demographic questionnaire. A food frequency questionnaire was used to investigate the diet group intake last year. Based on the normality of the data distribution, Independent t-tests and Mann-Whitney tests were used to compare quantitative variables between the two groups. Categorical variables were compared between cases and controls using the Chi-squared test. Logistic regression analysis was used to examine the possible association between food intake and aggression.

**Results:**

Compared to controls, aggressive men had significantly higher mean weight, height, and waist circumference (WC), p = 0.007, p = 0.001, and p = 0.043, respectively. After adjusting WC, energy intake, and educational level, in Model 1, intake of milk, cheese, poultry, red meat, legumes, egg, fruits, and vegetables had a significant protective role on the occurrence of aggression, (Odd Ratio (OR) = 0.36; 95% (Confidence Interval (CI) = 0.204, 0.670; P = 0.001), (OR = 0.440; 95% CI = 0.284, 0.781; P = 0.005), (OR = 0.621; 95% CI = 0.284, 0.781; P = 0.046), (OR = 0.358; 95% CI = 0.198, 0.647; P = 0.001), (OR = 0.434; 95% CI = 0.243, 0.773; P = 0.005), (OR = 0.411; 95% CI = 0.229, 0.736; P = 0.003), (OR = 0.332; 95% CI = 0.180, 0.614; P < 0.001), (OR = 0.310; 95% CI = 0.168, 0.572; P < 0.001), respectively.

**Conclusions:**

Lower WC and a diet containing high-quality protein, fruits, and vegetables can have a protective role against aggression and are recommended for men with an aggressive mood. This diet can affect plasma levels of tryptophan and, therefore, brain levels of serotonin.

## Background

Aggression is one of the most prevalent behavioral disorders that is often overlooked in men. Aggression is a behavior in which the intent is to harm another person, and is influenced by both personal and social factors [1]. Manifestations of aggression can be found in all groups of different cultures or locations [2]. The prevalence of male aggression worldwide is estimated to range between 20% and 68%. Aggression affects 24% of Brazilian men, 42% of South African men, and 46% of Asian men [3]. Direct and indirect manifestations of aggression can be observed. There are both verbal and physical forms of aggression. Verbal aggression may be accompanied a without physical assault [4].

The classic symptoms of depression, such as insomnia, boredom, and lethargy, often appear in more advanced stages in aggressive men. There is a difference between aggression and physical violence that is often misunderstood. Aggressive behavior is typically punishing, reprehensible, disciplinary, and hostile and often involves threats, insults, or even corporal punishment. Aggression can also lead to violence and physical aggression. An aggressive mood means protecting oneself in a way that violates the rights of others. This destructive behavior may be due to a lack of finances, emotional anxiety, or physical harm [4].

Several studies have demonstrated that lifestyle factors such as watching TV, stress, socioeconomic factors, and dietary factors affect aggression [5]. One study found that male prisoners who consumed more omega-3 (W3) were less aggressive [6]. Supplementing with essential vitamins, minerals, and fatty acids reduces antisocial behavior, including violence, according to another study [7]. Additionally, one research found that beverages containing aspartame, sodium benzoate, phosphoric acid or citric acid, high-fructose corn syrup, and caffeine might promote aggressive behavior in adolescents [8]. Consuming foods that are high in energy and low in nutritional value also increases the risk of mental disorders such as anxiety, aggression, and violent behavior in children and adolescents [9].

Due to the increasing prevalence of aggression in society, the damage caused by aggression to the individual and those around him, and the possible role of diet in aggression, in this study, we compared and evaluated the food intake and anthropometric measurements of aggressive and non-aggressive middle-aged men.

## Materials and methods

A total of 336 married middle-aged men (168 men with aggressive behaviors and 168 healthy controls), aged 35–55 years, participated in this case-control study. The eligibility criteria included middle-aged married men between 35 and 55 years of age who desired to participate in the study.

The appropriate sample size was 166 participants for each group based on one of the previous studies [10] and using the mean and standard deviation of protein intake (g/kg/day) in the case group (X1 ± SD1 = 1.86 ± 0.9) compared to the control group (X2 ± SD2 = 1.96 ± 0.88). The following formula based Kasiulevičius study [11], considering α = 0.05 (first type error) and β = 0.1 (second type error) was used:

$$n=\frac{{\left({{Z}_{1-\frac{\alpha }{2}}+ Z}_{1-\beta }\right)}^{2}\left({S}_{1}^{2}+{S}_{2}^{2}\right)}{{({\stackrel{-}{X}}_{1}-{\stackrel{-}{X}}_{2})}^{2}}$$

Finally, 168 subjects in each group and totally 336 subjects were included in the study. There was a 1:1 ratio between the case and control.

Subjects recurred among men working in the healthcare and education network who were referred to the psychology outpatient clinic in Shoushtar City, in Khozestan Province, from April 2022 to October 2022. Case ascertainment was assessed using the Buss-Perry Aggression Questionnaire (BPAQ). The case group comprised participants who scored above 29 on the BPAQ and worked in the healthcare and education network. The control group also consisted of men working in medical and educational centers who were colleagues of the case group.

The inclusion criteria were age between 35 and 55 years, marriage, diploma or higher, and diagnosis of aggressive mood by questionnaire (in the case group). Exclusion criteria included under- and/or over-reporting of dietary intake (energy intake outside the range of 800 to 4200 kcal per day), incomplete questionnaire answers, diabetes, hypothyroidism or hyperthyroidism, hypertension, heart disease, kidney disease, cancer, depression, and taking antidepressants, analgesics, or anti-obesity drugs. Age, occupation, marital satisfaction, and ethnicity were similar between the case and control groups.

The Medical Ethics Committee approved the study protocol at Shoushtar Faculty of Medical Sciences according to the 2013 Helsinki Declaration in November 2021 (Registration no: IR.SHOUSHTAR.REC. 1400.018). Before participating in the study, all participants signed an informed consent form. Information was collected using a general questionnaire regarding age, marital status, medical history, financial status, ethnicity, and level of education.

The BPAQ, revised in 1992, was used to assess aggression [12]. The questionnaire consisted of 29 questions and was graded on a five-point Likert scale ranging from 1 to 5. Among these 29 questions, four subscales representing physical aggression, verbal aggression, anger, and hostility. Scores for aggression were calculated by summing the subscale scores. The minimum and maximum scores are 29 and 145, respectively [13]. The Cronbach’s alpha coefficient was used to measure the internal validity of the scale. Physical aggression had an internal consistency of 0.82, verbal aggression of 0.81, anger of 0.83, and hostility of 0.8 [12]. The Hudson Marriage Satisfaction Questionnaire was used to measure marriage satisfaction [14]. In a study in the Iranian population, the reliability of the Hudson questionnaire was obtained 0.96 in women and 0.94 in men [15].

### Dietary assessment

During an extended face-to-face interview, a trained interviewer asked the participants about their dietary intake in the previous year. Routine food intake was recorded using a food frequency questionnaire (FFQ) designed based on the Iranian food pyramid. The reliability and validity of this FFQ confirmed in Iranian populations, previously [16, 17]. Moreover, interviewers used a booklet explaining household measures and the portion sizes for units of each food group [18]. Daily, weekly, monthly, yearly, or never frequency of consumption of each food group (based on the food pyramid) was recorded and converted to units per day. The foods included dairy products (milk, yogurt, cheese, and ice cream), bread and cereals (bread, rice, and pasta), meat, and alternatives (eggs, meat, and legumes), fruits, and vegetables. Further questions were asked about the type of dairy consumed (unprocessed and without preservatives dairy, processed low-fat dairy with less than 1.5% fat, processed medium-fat dairy with 2.5% fat, processed high-fat dairy with more than 2.5% fat), and the type of oil (butter, margarine, olive, canola, soy, and sunflower) consumed. As part of this questionnaire, the amount of fats and oils consumed, soft drinks (such as carbonated drinks and unnatural juices), and fast foods (such as pizza, sausages, sandwiches, and burgers), were also assessed and classified into low, medium, and high quantities, according to the amount consumed per week.

Anthropometric measures including their body height and weight of men were recorded, with an accuracy of 0.1 centimeters and 0.5 kg, respectively. Weight was measured without shoes or clothing and with only the minimum amount of clothing. Body mass index (BMI) was calculated by dividing the weight (kg) by height (m2). Additionally, using a measuring tape, waist circumference (WC) was determined between the lower border of the ribs and the iliac crest. All anthropometric measurements were performed three times, and the mean of the two closest measurements was calculated. We used the short form of the international physical activity questionnaire (IPAQ). Metabolic equivalents (METs) were calculated based on the participants’ usual physical activity. Participants were then divided into three groups according to their level of activity: low, medium, and high. Several countries have confirmed IPAQ’s validity and reliability [19]. In Iran, the validity and reliability of this questionnaire have been confirmed [20].

There were 368 subjects (183 cases, 185 controls) in the study, but 20 were excluded because of incomplete reporting by the questioners (12 cases, 8 controls). Nine controls and three cases were excluded because of over-reporting of food groups. The final analysis included 336 participants (168 cases and 168 controls).

### Statically analysis

SPSS statistical software (version 20) was used for data analysis. The normality distribution of all variables was evaluated using the Kolmogorov–Smirnov test. Quantitative variables with normal distribution are shown as the mean ± standard deviation (mean ± SD). Non-normal variables are also expressed as medians (25th and 75th percentiles). To compare quantitative variables between the two groups, an independent sample t-test or Mann-Whitney test was used, according to the normality of data distribution. The Chi-square test was used to compare qualitative variables between the case and control groups. The relationship between food consumption, and anthropometric indicators, and the risk of aggression was investigated using a multivariable logistic regression test. *P*-values < 0.05 was considered significant.

## Results

There were 347 enrolled subjects, of which 6 were excluded from the study due to incomplete reporting by the questioners. Additionally, two subjects from the case group and three from the control group were excluded due to the over-reporting of food groups. The final sample size was 336 participants (168 controls and 168 cases). Table 1 compares the demographic characteristics of the aggressive and control groups. The mean age of all participants was 35.65 ± 6.93 and the median of BMI was 26.08 (18.29, 34.98) kg/m^2^. There were no significant differences between the cases and controls in terms of mean age, BMI, level of satisfaction with married life, socioeconomic status (income), history of smoking, and type of job. The educational level of the aggressive group was lower than that of the control group (p = 0.015). In both the control and case groups, the median energy intake did not differ significantly [2732.4 (1587.2, 3946.1) kcal vs. 2852.5 (1426.3, 3845.3) kcal; respectively, p = 0.35].

**Table 1:** Comparing the characteristics of participants in the case and control groups based on their anthropometric indices

	Control (healthy) N = 168	Case N = 168	p-value
Age (years) ^£^	35.85 ± 7.65	35.33 ± 7.65	0.39
Weight (kg) ^£^	74.71 ± 9.61	81.25 ± 17.39	0.007^**^
Height (cm) ^€^	171.31 (151.0, 184.0)	174.30 (152.0, 194.0)	0.001^**^
BMI (kg/m^2^) ^€^	25.44 (18.29, 30.47)	26.66 (18.69, 34,98)	0.38
BMI category n (%)‡			0.055
< 25	84 (50)	74 (44.24)	
25-29.9	72 (42.56)	63 (37.5)	
> 30	12 (7.44)	31 (18.26)	
WC	87.23 (50.0, 109.0)	93.34 (59.0, 120.0)	0.043^*^
Central obesity n (%)‡	14 (8.5)	36 (21.2)	0.009^**^
Physical activity level n (%)‡			0.53
Low	78 (46.2)	73 (43.6)	
Medium	68 (40.7)	72 (42.8)	
High	22 (13.1)	23 (13.6)	
Aggression scores			
PA ^£^	20.106 ± 5.71	24.29 ± 7.97	0.021^*^
VA ^£^	11.29 ± 4.35	14.08 ± 4.27	0.040^*^
A ^£^	14.04 ± 4.95	20.95 ± 5.20	0.003^**^
H ^£^	17.31 ± 7.15	20.44 ± 7.77	0.015^*^
Marriage satisfaction (Hudsen score)	101.106 ± 12.15	103.89 ± 13.37	0.61
Smoking history n (%) ‡			0.83
Yes (Tobacco and cigarettes)	21 (12.8)	21 (12.5)	
No	147 (87.2)	147 (87.5)	
Income n (%) ‡			0.78
Lower-middle	68 (40.4)	62 (36.5)	
Middle	82 (48.9)	90 (53.8)	
Upper-middle	18 (10.6)	16 (9.6)	
Educational level n (%) ‡			0.015*
Junior-middle	71 (42.5)	97 (57.7)	
Senior and university	97 (57.5)	71 (42.3)	
Job n (%) ‡			0.12
Administrative jobs	77 (45.74)	56 (33.65)	
Self-Employed	91 (54.26)	112 (66.35)	

^£^ Data were analyzed using the independent sample t-test and expressed as mean (standard deviation)

^€^ Data are expressed as medians (25th, 75th percentiles) and analyzed using Mann-Whitney test

‡ The data are expressed as n (%) and analyzed by Chi-squared test

P-value < 0.05 was considered significant. *P < 0.05, ** P < 0.01, *** P < 0.001

Notes: BMI, body mass index; WC, Waist Circumference; PA, physical aggression; VA, verbal aggression; A, anger; H, hostility

**Fig. 1:**
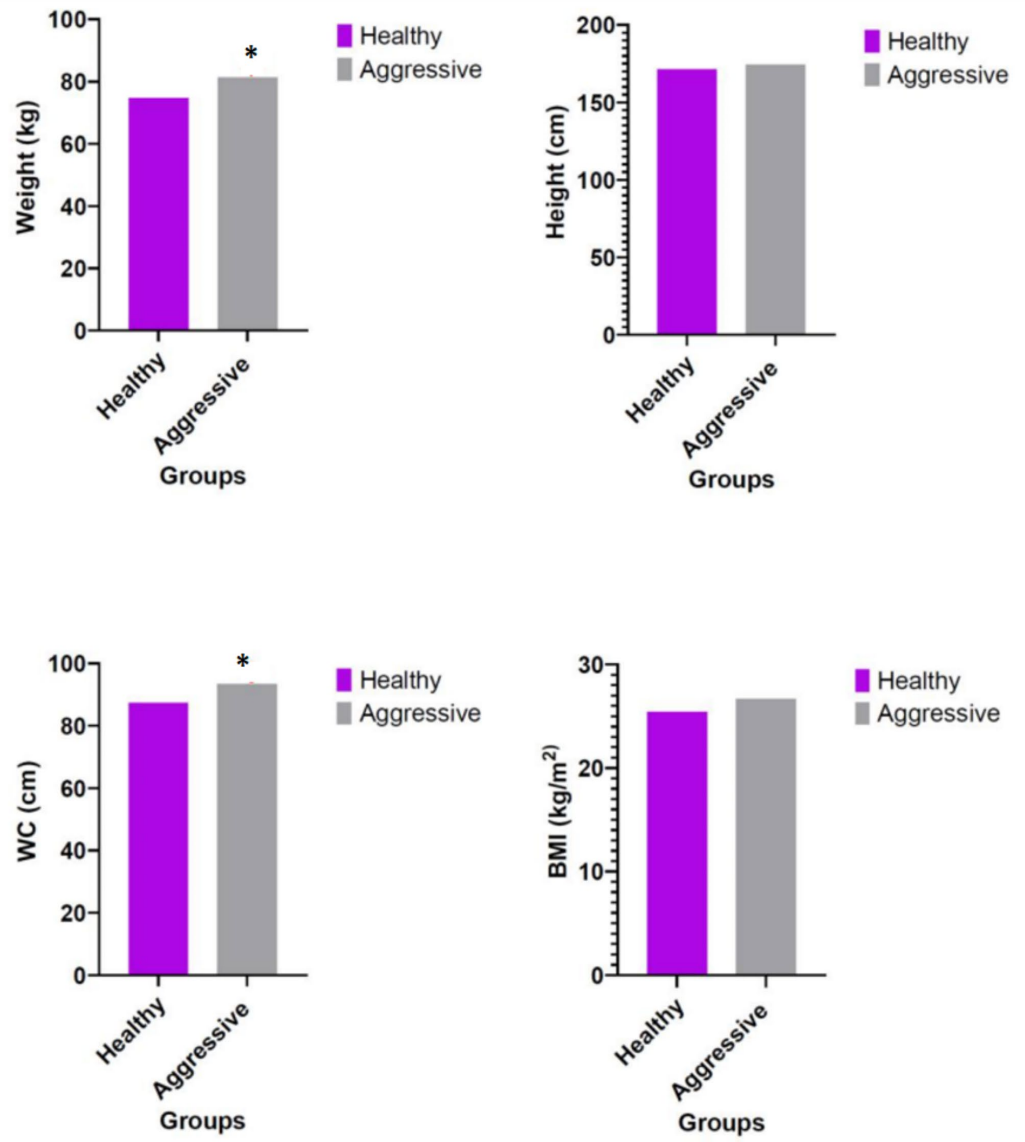
Comparison of anthropometric indices between the control and aggressive groups

As shown in Fig. 1, compared with the control group, aggressive men had significantly higher mean weight, height, and WC (p = 0.007, p = 0.001, and p = 0.043, respectively). Aggressive men also had a higher mean BMI, but this difference was not statistically significant (p = 0.382).

Table 2 provides the results of the logistic regression models regarding the association between food intake and aggressive behaviors, along with the odds ratios (OR) and 95% confidence intervals (CI). After adjusting WC, energy intake, and educational level, in Model 1, intake of milk, cheese, poultry, red meat, legumes, egg, fruits, and vegetables had a significant protective role on the occurrence of aggressive behaviors, (OR = 0.36; 95% CI = 0.204, 0.670; P = 0.001), (OR = 0.440; 95% CI = 0.284, 0.781; P = 0.005), (OR = 0.621; 95% CI = 0.284, 0.781; P = 0.046), (OR = 0.358; 95% CI = 0.198, 0.647; P = 0.001), (OR = 0.434; 95% CI = 0.243, 0.773; P = 0.005), (OR = 0.411; 95% CI = 0.229, 0.736; P = 0.003), (OR = 0.332; 95% CI = 0.180, 0.614; P < 0.001), (OR = 0.310; 95% CI = 0.168, 0.572; P < 0.001), respectively.

**Table 2:** Aggressive symptoms Odds ratios (ORs), based on food group intakes with 95% confidence intervals (CIs).

Daily food intake (per unit)	Crude OR	P-value	Adjusted OR Model 1	P-value
**Rice** (30 g cooked/ 1.05 Oz)	1.08 (1.03, 1.09)	0.004	1.04 (1.01, 1.07)	0.002
**Pasta** (30 g cooked/ 1.05 Oz)	1.82 (1.08, 3.06)	0.022	1.04 (1.02, 1.07)	0.001
**Bread** (30 g/ 1.05 Oz)	1.03 (1.01, 1.07)	0.042	1.04 (1.01, 1.07)	0.002
**Milk** (240 cc/ 8.12 Oz)	0.23 (0.08, 0.663)	0.006	0.36 (0.204, 0.670)	0.001^**^
**Yogurt** (180 cc/ 6.09 Oz)	1.09 (0.741, 1.60)	0.657	1.04 (1.01, 1.07)	0.001
**Dough (**Iranian traditional yogurt drink**)** (480 cc/ 16.2 Oz)	0.819 (0.536, 1.25)	0.355	1.050 (1.02, 1.07)	0.001
**Ice cream** (180 cc/ 6.09 Oz)	2.80 (1.246, 6.300)	0.013	1.044 (1.017, 1.072)	0.001
**Cheese** (45 g or 1.5 Oz)	1.66 (0.554, 5.394)	0.180	0.440 (0.284, 0.781)	0.005^**^
**Fish** (60–90 g/ 2–3 Oz)	1.91 (0.506, 7.254)	0.339	1.04 (0.91, 1.07)	0.170
**Poultry** (60–90 g/ 2–3 Oz)	0.456 (0.233, 0.893)	0.022	0.621(0.346, 0.931)	0.046^*^
**Red Meat** (60–90 g/ 2–3 Oz)	0.407 (0.178, 0.930)	0.033	0.358 (0.198, 0.647)	0.001^**^
**Legumes** (30 g cooked/ 1.05 Oz)	0.740 (0.150, 3.64)	0.711	0.434 (0.243, 0.773)	0.005^**^
**Egg** (one number)	1.342 (0.889, 2.0262)	0.161	0.411 (0.229, 0.736)	0.003^**^
**Fruits** (Apples, bananas, oranges, 1 A medium size number; Apricots, plums: 2–3 pcs)	0.575 (0.402, 0.823)	0.002	0.332 (0.180, 0.614)	< 0.001^***^
**Vegetables** (Cucumber, tomato, onion, etc., half a glass chopped)	0.596 (0.425, 0.837)	0.003	0.310 ( 0.168, 0.572)	< 0.001^***^

^§^OR calculated by *logistic regression* model. Weight, height, waist circumstance (WC), energy intake, and educational level were included in model 1 as covariates

As shown in Table 3, the group of men with aggressive behavior consumed a higher percentage of processed high-fat dairy. The aggressive group also had a higher percentage of cakes, sweets, and fast food. There were no significant differences in the consumption of soft drinks and the usual types of oils used between men with aggressive behavior and controls.

**Table 3:** Comparison of cake and sweets, soft drinks, fast food, dairy and oil types consumed between cases and control groups

Index		Total n = 336 Number (%)	(Controls) n = 168 Number (%)	(Cases) n = 168 Number (%)	*P*-value ^#^
	Unprocessed and without preservatives	133 (39.40)	75 (44.68)	58 (34.61)	
**Dairy type**	Processed low fat	47(14.14)	25 (14.89)	22 (13.46)	0.038*
	Processed medium fat	85(25.25)	43 (25.53)	42(25)	
	Processed high fat	71(21.21)	25 (14.90)	46 (26.93)	
**Cakes and sweets intake**	High	150 (46)	64 (38.29)	86 (51.40)	0.033*
	Medium	80 (23.73)	36 (21.27)	44 (25.96)	
	Low	106(30.27)	68 (40.44)	38 (22.64)	
**Soft drink**	High	57 (17.17)	25(14.89)	32 (19.23)	0.27
	Medium	77 (23.23)	30 (18.08)	47 (27.88)	
	Low	202 (59.60)	113 (67.03)	89 (52.88)	
**Fast food**	High	28 (8.58)	5 (3.19)	23 (13.46)	0.005^**^
	Medium	65 (19.62)	25(14.89)	40 (24.03)	
	Low	243 (71.8)	138 (81.92)	105(62.51)	
**The usual types of used oils**	Butter, margarine	129 (38.4)	64 (38.30)	65 (38.46)	0.10
	Olive, canola, soy, and sunflower	207 (61.6)	104 (61.70)	103 (61.54)	

^**#**^Data are expressed as n (%), and analyzed by *chi-squared* test

P-value < 0.05 was considered significant. *P < 0.05, ** P < 0.01, *** P < 0.001

## Discussion

This study examined the association between anthropometric measurements, dietary intake, and aggressive behavior among middle-aged married men. To our knowledge, no study has examined the relationship between food intake and aggressive behavior among middle-aged men. In our study, higher weight, height, and WC were observed in the aggressive group of men (p = 0.007, p = 0.001, and p = 0.043, respectively).

To ensure the same emotional and physical conditions among participants, married men were included in this study. In terms of satisfaction with married life, there was no significant difference between the case and control groups in terms of the Hudson score. Moreover, we recurred participants among men working in healthcare and education networks for similarities in education, job, and economy.

Vilchis et al. examined aggressive behaviors among obese and overweight school students in 2020 in a cross-sectional study. Similar to our findings, these researchers also reported a higher prevalence of aggression in overweight and obese participants [21].

This behavior is strongly modulated by steroid hormones. Androgens such as testosterone have been suggested to affect mood in men [22]. Weight gain and abdominal obesity impair sex hormone levels; thus, it has been hypothesized that middle-aged men with a larger WC will have higher androgen levels.

In our study, men with aggressive behavior consumed more processed high-fat dairy (p = 0.038), and fast food (p = 0.005). Clarke et al. also examined the effect of high-fat diet consumption on aggression in male rats in an experimental study and reported results similar to ours. Researchers have found that consuming a high-fat diet increases steroid hormone levels in mice and their aggressive behavior [23].

Furthermore, Zahedi et al., 2014 [9], concluded that adolescents who eat processed food are at a greater risk of psychological distress and aggressive behavior. It is known that junk food contains more total fat, saturated fatty acids, and trans fatty acids (TFAs). TFAs may contribute to aggressive behavior through several mechanisms such as changing the energy of cells [24], augmenting inflammatory damage [25], and oxidative stress [26]. Moreover, fast foods are especially deficient in W3, which is essential for brain function and mood [9].

In Model 1 of regression logistics, milk, cheese, poultry, red meat, legumes, eggs, fruits, and vegetables had protective effects against aggressive behavior after adjusting for weight, height, BMI, and education levels.

In our study, fruits and vegetables showed protective effects against aggressive behavior. Fruits and vegetables are good sources of fiber. In line with our study, in a recent research on adolescent girls, fiber intake was marginally associated with reduced odds of aggression [OR: 0.997, CI: (0.012, 0.999)] [10]. Furthermore, Khayatzadeh et al. (2019) reported that dietary intake of soluble and insoluble fiber is negatively associated with aggression in teenage girls [27]. Some possible mechanisms have been proposed to explain the link between fruits and vegetables and aggression. Fruits and vegetables are good sources of fiber. Dietary fiber reduces the glycemic index, affects brain chemistry, mood, and memory, and reduces the glycemic index. It has been shown that dietary fiber intake also influences the composition of gut microbiota, which may help to promote mental well-being [28].

We observed that milk, cheese, poultry, red meat, and legumes had protective effects against aggressive behavior.Tryptophan is abundant in milk, cheese, poultry, red meat, legumes, and eggs, and affects plasma levels of tryptophan and, therefore, brain levels of serotonin [29]. Tryptophan is required for the synthesis of the neurotransmitter serotonin (5-hydroxytryptamine). Dietary tryptophan deficiency may contribute to serotonin depletion in the central nervous system (CNS) and cause self-mutilation and aggression [30, 31]. The intake of tryptophan-rich foods can help regulate serotonin synthesis in the CNS.

### Strength and limitation

To the best of our knowledge, the relationship between food group intake and the risk of aggression among middle-aged men has not yet been published. Additionally, we used logistic regression to reduce the effects of potential cofactors. The present study has some limitations. First, this study was a case-control study, and it could not demonstrate causality between dietary intake and aggression. Second, owing to the retrospective nature of the FFQ questionnaire, recall bias could exist despite the use of a validated FFQ. Finally, we adjusted the effects of some potential cofactors, but may still have ignored some unmeasured cofactors.

## Conclusions and implications for research and practice

In our study, men in the aggressive group had higher weight, height, and WC. Aggressive men may have a better mood and controlled aggression, in the normal BMI and WC ranges. To date, there have been no clinical trial studies in this field, but it seems that maintaining a BMI in the normal range (18.5 to 24.9) is appropriate. Moreover, a diet containing meat (high-quality protein), fruits, and vegetables may protect against aggression. Vegetables and fruits contain fibers with a lower glycemic index. Fibers in fruits and vegetables can reduce the glycemic index and affect brain chemistry, mood, and memory. Moreover, lower consumption of processed high-fat dairy and fast food can have a beneficial effect on weight, WC control, and emotion in men with aggressive behavior. Observing a significant relationship between anthropometric indicators and the consumption of some food groups can be a policy for future studies and is strongly recommended. The findings of our study needed that supported by prospective studies and clinical trials.

## Data Availability

The data that support the findings of this study are available upon request from the corresponding author, SA. The data are not publicly available due to restrictions for example containing information that could compromise the privacy of research participants.

## Abbreviations

FFQ: Food frequency questionnaire
BMI: Body mass index
WC: Waist Circumference
OR: Odds ratio
CI: Confidence interval.
